# Dermatomyositis presenting with rhabdomyolysis and acute renal failure; an uncommon manifestation

**DOI:** 10.4103/0972-2327.48853

**Published:** 2009

**Authors:** Deepika Joshi, Niraj Kumar, Anand Rai

**Affiliations:** Department of Neurology, I.M.S., B.H.U., Varanasi-221005, India

**Keywords:** Acute tubular necrosis, dermatomyositis, myoglobinuria, rhabdomyolysis, renal failure

## Abstract

Rhabdomyolysis and myoglobinuria are a rare complication of dermatomyositis. Such patient can land up in acute renal failure. Recognition of this fact has important therapeutic implications as patients require immunotherapy in addition to the symptomatic treatment for renal failure. We report a case of dermatomyositis with evidence of rhabdomyolysis and myoglobinuria presenting with acute renal failure. The patient responded dramatically to corticosteroid therapy.

## Introduction

Dermatomyositis (DM) is an idiopathic inflammatory myopathy with characteristic cutaneous findings.[1] Myoglobinuria results from extensive muscle fibre necrosis and may be caused by traumatic causes or nontraumatic causes such as infection, drugs or metabolic myopathies. Both polymyositis and dermatomyositis are rare causes of rhabdomyolysis, which occurs because of ongoing muscle fibre destruction. Recognition of this fact has important therapeutic implications as patients can land up in renal failure. We report here an interesting case of severe acute dermatomyositis presenting with rhabdomyolysis and renal failure.

## Case Report

A 20-year-old female patient presented with history of acute onset quadriparesis of 10 days duration and decreased urine output of 3 days duration. Her complaints started in the form of throbbing pain in the right calf region which spread to whole of right lower limb in one day.This was not associated with fever, trauma to the leg or back. Next morning, she noticed similar pain in left calf region which involved whole of left lower limb in one day. Simultaneously, patient also developed tingling sensation in both lower limbs. After 2 days of onset, she noticed proximal muscle weakness in both lower limbs. Over the next 3 days, weakness progressed to involve the whole lower limb. Along with the lower limb involvement, she also noticed pain and proximal weakness in both upper limbs which progressed over next 3 days to involve the whole upper limb. After 1 week of onset she developed complete weakness of both upper and lower limbs along with neck and truncal muscle weakness without any respiratory, bulbar involvement, extraocular or facial muscle involvement.

On the 8^th^ day of illness, she started vomiting (3 to 4 episodes per day) and developed oliguria and edema over face and feet. At this point of time, she was admitted in the nephrology department of our hospital and investigated accordingly.

Investigations revealed Hb – 10.2gm%, TLC – 13,800/cu mm, DLC – P69, L28, E1, M2, ESR – 44 mm in 1st hour (Westergreen method), platelet count – 254000/cu mm, reticulocyte count –1.8%, blood urea – 283mg%, serum creatinine – 8.6mg%, serum sodium – 131.9 mmol/L, serum potassium – 8.09 mmol/L, serum calcium – 6.3mg%, serum phosphorus – 7.6mg%, AST – 664 IU/L, ALT – 488 IU/L, ALP – 241 IU/L, serum total bilirubin – 0.5mg%, serum direct bilirubin – 0.4mg%, serum total protein – 5.2gm%, serum albumin – 2.7gm%, serum creatine phosphokinase – 28,950 IU/L (Normal value – 24–90 IU/L).

Urine was brown colored (coca-cola colored) and microscopic examination revealed protein-+1, granular casts – 3–4/HPF; no bile pigments, bile salts, occult blood, and ketone bodies. Urine was positive for myoglobin (750 ng/L). Immunological screening like antinuclear antibodies, rheumatoid factor and C-reactive protein were normal. Screening for human immunodeficiency virus and hepatitis B surface antigen, anti-HCV were negative (Other specific autoantibodies were not done due to financial constraints). Chest X-ray and abdominal ultrasound were normal.

She was being treated by the nephrologists as a case of severe rhabdomyolysis with acute renal failure and underwent one sitting of peritoneal dialysis and six sittings of hemodialysis over the next 15 days. There was minimal biochemical improvement, and in addition to the persistent weakness, the patient developed complaints of breathlessness for which a neurology consultation was sought.

General examination revealed normal temperature, pulse rate of 90/min and a blood pressure of 130/90 mmHg. Respiratory rate was 32/min, and single breath count was 18. Chest auscultation was normal. Patient was anemic and had bilateral pitting pedal edema and facial puffiness. There was no lymphadenopathy, pallor, cyanosis, icterus, or clubbing. A reddish brown rash was noted over anterior aspect of chest wall and back, with Gottron's papules over the knuckles. On detailed questioning, she gave a past history of polyarthritis about 8–10 months back.

Neurological examination revealed neck muscle and truncal muscle weakness, hypotonia in all four limbs and marked weakness in proximal (1/5) and distal (2/5) group of muscles in both upper and lower limb muscles. These muscles were tender. Sensory system examination was normal. Deep tendon reflexes were diminished and plantars were not elicitable.

Electrophysiological study revealed reduced amplitudes over motor nerves in both upper and lower limbs. Sensory nerve conduction studies were normal. Electromyography revealed increased insertional activity, profuse fibrillations and positive sharp waves with low amplitude, polyphasic, short duration potentials.

Muscle biopsy of proximal muscle (left vastus lateralis) showed extensive myonecrosis and active rhabdomyolysis. There was striking sectorial involvement in each funicle with brisk myophagocytosis, several regenerating clusters and fibrosis. Some epi- and perimyseal vessels revealed sparse perivascular inflammation. Some fascicles revealed perifascicular atrophy. Focal fibrosis was also seen.

Based on the characteristic rash with muscle weakness and biopsy features, a diagnosis of dermatomyositis with myoglobinuria and renal failure was obtained. She was given high doses of methylprednisolone 1 gm/day for only 5 days followed by 1.5 mg/kg/day. She had a dramatic clinical response with improvement in muscle power and respiratory symptoms noted within 5–7 days of starting treatment. Biochemical parameters also improved, and with two weeks of steroid treatment, her urine output increased to 1500 ml per day and muscle power improved to 4-/5, with marked reduction in muscle tenderness. In the biochemical parameters, there was a marked improvement with blood urea becoming 58mg%, serum creatinine – 1mg%, serum CPK – 202I U/Lt, serum sodium – 138 mmol/Lt, serum potassium – 3.5 mmol/Lt, and urine myoglobin – 50 ng/L.

## Discussion

Thus, this patient with clinical features of dermatomyositis went on to develop myoglobinuria due to ongoing rhabdomyolysis and landed in renal failure.

This patient had the characteristic rash, muscle weakness, electrophysiological features suggestive of a myositis and focal features of muscle inflammation and necrosis, with perifasicular atrophy consistent with a diagnosis of dermatomyositis. The patient had a dramatic response to immunotherapy with steroids, which is a feature of inflammatory muscle disease.

Myoglobinuria is usually the result of rhabdomyolysis or muscle destruction. [Table 1] Any process that interferes with the storage or use of energy by muscle cells can lead to myoglobinuria. The release of myoglobin from muscle cells is often associated with an increase in levels of creatine kinase (CK), aldolase, lactate dehydrogenase (LDH), serum glutamic-oxaloacetic transaminase (SGOT) and serum glutamic-pyruvic transaminase (SGPT). When excreted into the urine, myoglobin, a monomer containing a heme molecule similar to hemoglobin can precipitate, causing tubular obstruction and acute renal insufficiency.

**Table 1:** Causes of rhabdomyolysis giving rise to myoglobinuric acute renal failure

Trauma and compression	Crush injury, burns, electric shock injury
Ischemia	Vascular occlusion, immobility/ compression {coma, substance abuse [alcohol, heroin], anesthesia}, sickle cell disease, air embolism.
External myolysis:	Exertional myoglobinuria occurs in most athletes, convulsions, delirium tremens.
Viral myositis	Viral infections from a wide variety of organisms can cause myositis and myoglobinuria Children with influenza A and influenza B viral infections can present with tenderness in calves and lower extremities. Treatment is generous hydration to facilitate myoglobin excretion.
Electrolyte disorders:	Potassium depletion has been particularly associated with myoglobinuria.
Toxins, drugs, and diet	Snakebites and other venoms can cause muscle necrosis and myoglobinuria. Alcohol, cocaine, amphetamines, phencyclidine, ecstasy, ethylene glycol, isopropyl alcohol, phencyclidine, AZT and lovastatin has been associated with myoglobinuria.
Infection or sepsis syndromes:	Syndromes involving muscle destruction include gas gangrene, tetanus, Legionnaire disease, or shigellosis. Coxsackie viral infections with myositis may be the most common cause of mild myoglobinuria.
Endocrine disorders:	Diabetic ketoacidosis, myxedema, and nonketotic hyperosmolar comas can disrupt muscle energy.
Malignant hyperthermia and high fevers: Metabolic Disorders	Patients with defects of carbohydrate metabolism (e.g. myophosphorylase, phosphofructokinase, phosphohexoisomerase deficiency) have symptoms of easy fatigability or cramping induced by dynamic isometric exercise, such as heavy lifting, or prolonged exercise, such as swimming or running. Acute muscle breakdown can lead to myoglobinuria. These patients typically present after participating in high-intensity exercise, such as weight lifting. Defects in lipid metabolism include carnitine deficiency, beta-oxidation enzyme deficiency, or disorders of fatty acid transport. Prolonged fasting or prolonged activity induces muscle pain and myoglobinuria. Fever, sepsis, and exposure to cold can also induce muscle fatigue in this set of disorders. These patients typically develop symptoms after prolonged low-intensity exercise, such as walking. Patients with mitochondrial disorders (beta-oxidation disorders) usually present with static and progressive muscular weakness.
Heat exhaustion and cold exposure	These conditions induce abnormal muscle metabolism by means of various mechanisms, including poor perfusion and decreased oxygenation, acidosis, rhabdomyolysis, or glucose and/or glycogen
Inflammatory muscle disease	Polymyositis, dermatomyositis

There have been case reports of dermatomyositis with myoglobinuria or renal failure with variable treatment outcomes both with and without malignancy,[2–8] Renal involvement in patients with polymyositis (PM)/dermatomyositis (DM) was previously thought to be uncommon, but two main types of renal lesion have been described.[8] First, acute tubular necrosis with renal failure related to myoglobulinemia and myoglobulinuria is a well-recognized feature of acute rhabdomyolysis. Second, chronic glomerulonephritis has been infrequently reported in a small group of patients with PM/DM. A retrospective study analysed the records of 65 patients of polymyositis and dermatomyositis for investigating the incidence, severity and prognosis of renal disease in PM/DM patients admitted to a single centre over a 10-year interval. Of the 65 patients, 14 were found to have suffered varying degree of renal involvement, and the incidence rate was 21.5%. All the 14 patients had varying degree of haematuria and proteinuria. Acute tubular necrosis with renal failure developed in four patients with PM and in five patients with DM. Renal biopsy in two DM patients with overt proteinuria revealed IgA nephropathy in one and membranous nephropathy in the other. The authors therefore concluded that renal involvement in PM/DM patients is not as uncommon as previously thought.[9]

Our patient could also have developed acute tubular necrosis which improved over two weeks, but the development of the characteristic rash with muscle weakness and muscle biopsy features of inflammation with a dramatic improvement in muscle weakness with steroids points to an underlying inflammatory muscle disease (DM). Recognition of this fact is important as this has therapeutic implication for starting immunotherapy in addition to the symptomatic dialysis, alkalinization and hydration treatment for rhabdomyolysis, as is evident in our case.
